# Biodegradation Efficacy of *Aspergillus niger* and *Trichoderma harzianum* on Low-Density Polyethylene

**DOI:** 10.3390/polym17101303

**Published:** 2025-05-10

**Authors:** Momina Ahmed, Shazia Iram, Noshabah Tabassum, Mahnoor Sajid, Kingkham Paseutsakoun, László Aleksza, András Székács

**Affiliations:** 1 Department of Environmental Sciences, Fatima Jinnah Women University, The Mall, Rawalpindi 46000, Pakistan; mominaahmed857@gmail.com (M.A.); noshabatabassum@fjwu.edu.pk (N.T.); mahnoorsajid75@gmail.com (M.S.); 2 Institute of Environmental Sciences, Hungarian University of Agriculture and Life Sciences, Páter Károly u. 1, H-2100 Gödöllő, Hungary; paseutsakoun.kingkham@phd.uni-mate.hu (K.P.); aleksza.laszlo@uni-mate.hu (L.A.); 3 Profikomp Environmental Technologies Inc., Kühne Ede u. 7, H-2100 Gödöllő, Hungary

**Keywords:** polyethylene, microbial degradation, fungal strains, *Aspergillus niger*, *Trichoderma harzianum*

## Abstract

This study investigates the biodegradation potential of two fungal strains, *Aspergillus niger* and *Trichoderma harzianum*, on polyethylene plastic bags, addressing the environmental challenges posed by the resistance of the plastic material to degradation. The fungi were cultivated, and their spore suspensions were tested for polyethylene degradation in both the soil and liquid salt media. Degradation was assessed using weight loss measurements, thermogravimetric analysis (TGA), Fourier-transform infrared spectroscopy (FTIR), and scanning electron microscopy (SEM). After one month in liquid medium, *A. niger* induced a 45.62 ± 0.21% weight loss of polyethylene, while *T. harzianum* achieved a 36.0 ± 0.21% weight reduction. In soil, weight losses of 9.09 ± 0.08% and 10.00 ± 0.18% were observed after two months, respectively. TGA confirmed that the fungus-treated polyethylene samples were less thermally stable than untreated controls, indicating successful biodegradation. FTIR analysis revealed structural changes in the degraded polyethylene, while SEM images demonstrated significant surface alterations, including pitting, roughening, cracks, holes, and fungal colonization. These findings confirm the enzymatic action of fungi in degrading polyethylene into monomeric forms. The study highlights the potential for fungal biodegradation as an environmentally friendly strategy to mitigate plastic pollution. Future studies should characterize the specific enzymes involved and explore genetic engineering to enhance degradation rates.

## 1. Introduction

Global plastic production has grown from 50 million metric tons in 1976 to 413.8 million metric tons in 2023 [1]. Concurrently, the amount of plastic waste generated worldwide was estimated at 353 million metric tons in 2019 [2]. Common synthetic polymers, such as polystyrene, polyethylene, polyvinyl chloride, polypropylene, and polyethylene terephthalate are widely used in household products and packaging due to their affordability, lightweight nature, and durability. However, their extensive use significantly contributes to plastic waste accumulation in the environment.

Over time, the degradation of these polymeric materials has become a growing concern. The amount of macroplastics polluting the environment globally was estimated at 21 million tons in 2022, representing an increase of over 30% compared to the previous decade. Plastics contaminate not only our soils and the hydrosphere, but also contributing 3.8% of total global greenhouse gas emissions [3].

In plastic waste management, material recycling takes priority, followed by chemical recycling (pyrolysis) and biodegradation [4,5]. Biodegradation—the breakdown of polymers by the metabolic activities of microorganisms like fungi, bacteria, and algae—offers an efficient and sustainable solution to plastic waste. This aerobic process involves several stages. The first stage is biodeterioration, where microbes initiate the degradation process by attacking the surface of the plastic, leading to alterations in its chemical and physical properties. This is followed by biofragmentation, where microbial enzymes, including ureases, esterases, and proteases, cleave the polymer chains, breaking them into smaller fragments [6]. In the third stage, termed assimilation, these fragments are consumed by microorganisms. Finally, in the mineralization step, the plastic material within the microbial cells undergoes further oxidation, converting these into byproducts like CO_2_, CH_4_, H_2_O, and H_2_S [7]. This natural biodegradation process presents a promising avenue for mitigating the environmental impacts of plastic waste by harnessing microbial activity to break down synthetic polymers.

Fungi can thrive in a wide range of natural and artificial habitats, colonizing various matrices, including soil, water, and air. They are incredibly adaptable organisms [8,9]. The biodegradation of plastics involves fungal growth on the plastic surface, where fungi utilize the plastic as a substrate, influenced by environmental factors such as pH and temperature. Various fungal strains have been demonstrated to metabolize plastic polymers as their sole carbon source, thereby degrading them [10,11,12,13]. Primarily, the soil-borne fungi of the phylum Ascomycota—such as *Alternaria*, *Aspergillus*, *Cladosporium*, *Fusarium*, *Penicillium* spp.—are among the most effective plastic degraders. Interestingly, marine fungi have also been identified with plastic-degrading capabilities [14,15]. For instance, *Parengyodontium album*, a marine fungus, has been shown to be capable to degrade polyethylene, suggesting that marine fungi could be important degraders of complex organic matter in marine environments [16].

Fungi utilize a wide range of enzymes, predominantly hydrolases (EC 3), including esterases (EC 3.1), cutinases (cutin hydrolase, EC 3.1.1.74), lipases (EC 3.1.1.3), pectinase (EC 3.2.1.15), proteases (EC 3.4), and ureases (urea amidohydrolase, 3.5.1.5). They also employ oxidoreductases (EC 1) such as laccases (EC 1.10.3.2) or peroxidases (EC 1.11.1) [10,17,18] to facilitate plastic degradation. This extensive enzymatic repertoire enables fungi to decompose virtually all chemical classes of plastics [12], albeit with varying efficiencies and rates. However, fungal strains exhibit specific preferences for different plastic types [11]. Numerous species have demonstrated the ability to degrade polyethylene, including *Fusarium* [19,20,21,22,23], *Aspergillus* [10,19,20,21,24,25,26,27,28,29,30,31,32,33,34,35], *Trichoderma* [26,36,37,38,39,40], *Alternaria* [41], *Penicillium* [20,42,43], *Candida* or *Acremonium* [20,35,44], *Rhizopus* [45] *Cladosporium* [46,47], or other species [10,20,33,41,44,48], as well as fungal consortia [49]. Polyethylene’s lack of functional groups that are easily cleavable by hydrolases makes its decomposition challenging. Nonetheless, its degradation largely depends on various abiotic and biotic factors [22] and primarily involves laccase enzymes that promote alkyl chain depolymerization [37,38,50,51,52], verified not only by degradation monitoring but also in in silico molecular modeling [53].

The microbial degradability of polyethylene varies based on structural features such as polymer crystallinity. Low-density polyethylene (LDPE), characterized by a higher degree of branching, exhibits lower crystallinity compared to high-density polyethylene [22], rendering it more susceptible to microbial degradation.

Various additives, acting as enzymatic mediators, have been shown to enhance the efficiency of polymer degradation [51]. Notably, monolayer-forming hydrophobins from *Trichoderma* species have been found to significantly improve the enzymatic hydrolysis of polyethylene terephthalate when fused to cutinase enzymes [54,55,56]. *Trichoderma* strains are well known for their beneficial effects on plant health [57]. Interestingly, the application of Mater-Bi bioplastic granules alongside these fungi has been observed to further promote plant growth, suggesting a synergistic interaction between the bioplastics and the fungi [58]. Additionally, a combination of *A. niger* and *T. harzianum* has been successfully applied to decompose polyethylene terephthalate, indicating the potential of fungal consortia in plastic biodegradation efforts [59]. However, the degradative activity of *Trichoderma* species can be influenced by environmental factors. Exposure to herbicides such as metolachlor has been found to reduce the fungal degradation capabilities. Conversely, the presence of LDPE microparticles did not adversely affect *T. harzianum* strains resistant to this herbicide [39,60,61]. Kunlere et al. [29] reported evidence of LDPE biodegradation by two fungal species, *A. flavus MCP5* and *A. flavus MMP10*, demonstrating that *A. flavus* can utilize LDPE as both a nitrogen and carbon source in the absence of additives. Although LDPE is highly resistant to biodegradation due to its long carbon chains, fungal species from the genus *Aspergillus* can grow freely and abundantly in soil and waste environments. Additionally, because *Aspergillus* species take longer to incubate than other fungi, they are commonly used in LDPE biodegradation.

This study evaluated the biodegradability of polyethylene by *T. harzianum* and *A. niger*, two well-known fungal strains, by systematically investigating their synergistic interaction for LDPE biodegradation. Unlike prior research, which often relied on controlled laboratory conditions, the present study simulates real-world environmental conditions (e.g., soil and aquatic systems) to evaluate fungal biodegradation. The study demonstrates that *A. niger* and *T. harzianum* employ distinct mechanisms and exhibit varying efficacies in degrading polyethylene, contingent upon environmental media (liquid and soil). *A. niger* primarily utilizes extracellular enzymes, notably lipase and esterases, to facilitate the more intensive polyethylene degradation in liquid media than in soil. Conversely, *T. harzianum* primarily employs a biofilm-mediated oxidative breakdown pathway that is more dominant in soil than in liquid media. By tailoring the application of these fungi according to given habitats (e.g., *A. niger* for aquatic systems, *T. harzianum* for terrestrial waste), this environment-dependent specialization reinterprets bioremediation techniques. The results offer a novel framework for maximizing fungal-based plastic breakdown in a variety of real-world settings.

## 2. Materials and Methods

### 2.1. Materials

**Figure 1:**
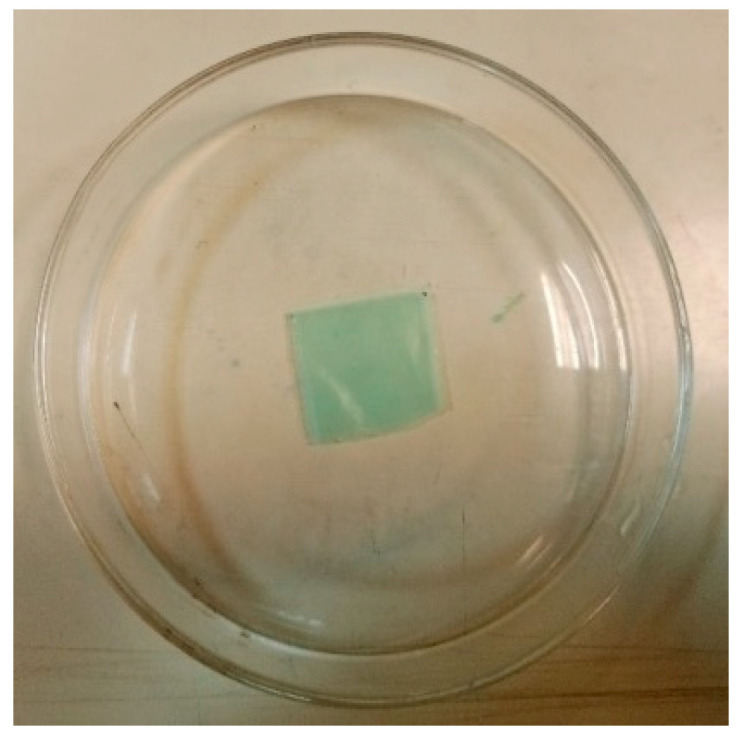
A polyethylene film strip sample (size: 2 × 2 cm, thickness: 1 mm) applied in the experiments.

LDPE, as the polymer material of standard quality polythene shopping bags, the most common packaging material used in shopping malls in Pakistan, was used in the present study. Manufacturer-provided, previously unused polyethylene plastic bags were cut into strips of size: 2 × 2 cm, thickness: 1 mm. These strips were sterilized by soaking in 70% ethanol and oven-dried for 24 h prior to use in the degradation experiments [62] (Figure 1).

Farmland soil was collected and sterilized using formaldehyde to create controlled conditions for the degradation assays. Soil pots were autoclaved in a single cycle before the commencement of the experiment to ensure a sterile environment for fungal growth.

The mineral salt medium for the microbial decomposition experiments was prepared according to the methodology outlined by Chaudhary et al. [62]. The mineral salt concentrations were 0.2 g L^−1^ (NH_4_)_2_SO_4_, 0.04 g L^−1^ KH_2_PO_4_, 0.5 g L^−1^ K_2_HPO_4_, 0.1 g L^−1^ NaCl, 0.02 g L^−1^ MgSO_4_·7H_2_O, 0.001 g L^−1^ FeSO_4_, and 0.002 g L^−1^ CaCl_2_·2H_2_O. All chemicals used in this research were of analytical grade, with 99.99% purity, and were utilized without further purification. Deionized water was used for all the experiments.

### 2.2. Cultivation of Fungal Strains

**Figure 2:**
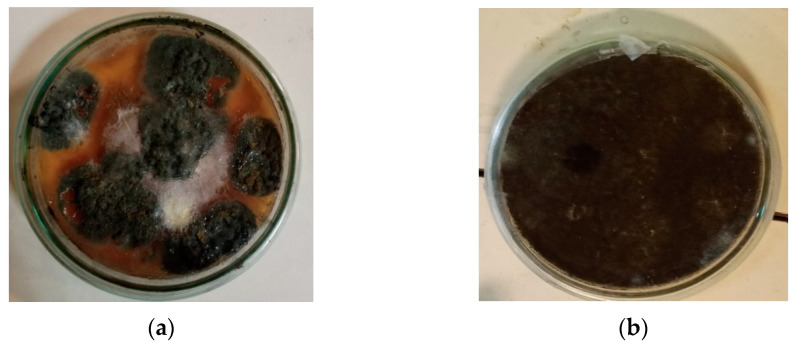
Fresh and thick colonies of *Aspergillus niger* (**a**) and *Trichoderma harzianum* (**b**).

Two fungal strains, *A. niger* and *T. harzianum*, were received from the Environmental Mycology and Ecotoxicology Laboratory, Fatima Jinnah Women University, Rawalpindi, Pakistan. The strains were selected from the fungal strain collection of the laboratory, obtained by the classical isolation and identification approach of fungal pathogens based on colony morphology—including the color, texture, and pattern—and microscopic examination of spore characteristics [63,64]. The strains were revived on potato dextrose agar (PDA) medium, prepared by dissolving 19.5 g of PDA in 500 mL distilled water. The medium was sterilized by autoclaving at 121 °C for 10 min, then poured into Petri dishes and allowed to solidify for 24 h. Freeze-dried fungal cultures were inoculated onto the solidified medium and incubated at 30 °C for five days. To maintain pure fungal colonies, plates were refreshed weekly [65]. Upon the completion of sporulation (Figure 2), spores were harvested by adding 10 mL of distilled water to each Petri dishes, followed by the gentle scraping of the spores with a sterile loop. The resulting spore suspension was vortexed for 10 min to ensure the uniform distribution and then diluted to a known volume with distilled water for subsequent experimental procedures [65].

### 2.3. Polyethylene Degradation Assay in Liquid Medium and Soil

In the liquid degradation assay, 50 mL of the prepared mineral salt solution was added to each flask, along with two polyethylene strips and 10 mL of fungal spore suspension. The flasks were incubated in a shaker at 150 rpm for one month. Control samples consisted of polyethylene strips in minimal salt medium without fungal inoculum [66]. For the degradation assay in soil, polyethylene strips were buried in sterilized soil pots, and 10 mL of fungal spore suspension was added to each pot. The pots were incubated in dark conditions for two months. Control pots consisted of polyethylene strips buried in autoclaved soil without fungal inoculum [66]. All degradation assays were performed in triplicates in both media.

### 2.4. Monitoring of Environmental Variables

During the incubation period, environmental variables such as temperature and pH were monitored weekly. Temperature was measured using a standard thermometer. Soil pH was assessed by extracting soil samples from each pot, mixing them with purified water in a 1:1 ratio, and measuring the pH of the supernatant using a potentiometer. The pH of the liquid medium was also monitored weekly [67]. Variations in pH within culture media serve as indicators of the metabolic activity of fungal strains [43]. In this study, pH changes in each fungal medium were monitored at seven-day intervals throughout the incubation period.

After the incubation period, samples were harvested by washing with 70% ethanol, followed by oven drying. Weight loss was determined in triplicates to quantify degradation. The initial weight of the polymer samples was recorded prior to exposure to the fungal cultures. Post exposure, the plastic samples were removed, cleaned with ethanol, and dried overnight at 60 °C. Weight loss was then determined using the formula:

(1) $$Weight\;loss\%=\frac{M_{0}-M_{f}}{M_{0}}\times 100$$

where M_0_ is the initial weight of the LDPE sample, and M_f_ is the final weight of LDPE after biodegradation [27].

### 2.5. Instrumental Analysis

Chemical and surface analyses were conducted using Fourier transform infrared spectroscopy (FTIR), thermogravimetric analysis (TGA), and scanning electron microscopy (SEM) to assess the physical and chemical changes in the polyethylene samples [66]. FTIR spectroscopy was performed using an FTIR-8400 instrument (Shimadzu Corp., Kyoto, Japan) to identify the chemical structural changes in the polymer samples after the incubation period [43]. Sample strips were prepared by gently sanding the surface with fine-grit sandpaper to remove contaminants, followed by cleaning with 70% ethanol, and lint-free wipes. For FTIR analysis, samples were pressed directly onto the spectrometer sample holder with uniform clamping force. FTIR spectra of the treated samples were compared to those of the untreated controls with a resolution of 4 cm^−1^ and Happ–Genzel apodization.

TGA was conducted using a TGA 8000 thermogravimetric analyzer (PerkinElmer Inc., Shelton, CT, USA) to evaluate the thermal stability of the samples. Approximately 5–10 mg of each sample was precisely weighed and loaded into alumina crucibles. Thermal stability was assessed under a temperature range of 30 to 500 °C, with a heating rate of 10 °C min^−1^, and a nitrogen atmosphere maintained by a flow of 100 cm^3^ min^−1^ [68,69].

SEM analyses were performed using a Vega-3 LMU instrument (Tescan Korea Ltd., Seoul, Republic of Korea). Since the samples were not conductive, they were gold-plated in a sputter coater prior to the SEM investigation to enhance conductivity. Subsequently, the samples were placed inside the thermionic emission chamber. Backscattered electron images were collected to study the surface morphology of the polyethylene samples. A magnification rate of 1000× was used to capture the detailed images of the LDPE surface [30].

## 3. Results

### 3.1. Polyethylene Degradation Assay by Weight Loss

The weights of the polyethylene samples were measured before and after incubation with the fungal cultures. Weight loss percentages were calculated using the formula outlined in Equation (1) and summarized in Table 1. No weight loss (0% weight loss) was observed in the untreated control experiments (polyethylene strips without fungal inoculum). In the liquid medium, *A. niger* exhibited a significantly higher percentage weight loss the percentage weight loss (45.62 ± 0.21%) compared to *T. harzianum* (35.95 ± 0.18%), with a two-tailed *t*-test yielding *p* < 0.0001. Conversely, in soil, *T. harzianum* demonstrated a marginally but significantly higher degradation (10.00 ± 0.19%) than *A. niger* (9.09 ± 0.08%), with *p* = 0.006 (*p* < 0.01).

**Figure 3:**
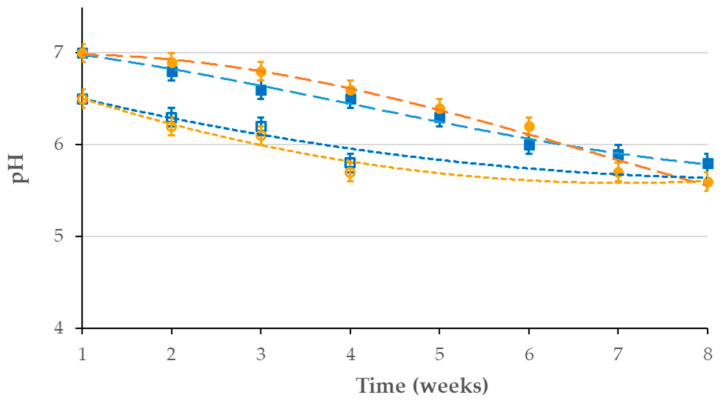
The weekly monitoring of changes in pH during biodegradation by *Aspergillus niger* (blue signs and lines) and *Trichoderma harzianum* (orange signs and lines) in liquid medium (hollow signs and dotted lines) and in soil assembly (filled signs and dashed lines).

The initial pH of the liquid medium was adjusted to approximately 6.5 for both fungal isolates. Throughout the incubation period, the pH of the medium decreased weekly. After one month, the pH of the medium incubated with *A. niger* was measured at 5.8, while that of *T. harzianum* was 5.7. In soil, the initial pH was recorded at 7.0 prior to incubation with the fungal cultures. During the degradation period, the soil pH also decreased. After incubation, the pH of the soil treated with *A. niger* was 5.8, whereas the soil with *T. harzianum* recorded a pH of 5.6 (Figure 3). These pH variations in the culture media indicated that the pH in the media and the degradation rates of polyethylene are interdependent as fungal enzymes work best at pH 5–7, while acid secretion (e.g., oxalic acid) can accelerate oxidation. The pH may drop due to the emergence of organic acids and polyethylene breakdown byproducts, but extreme acidity may inhibit those enzymes. The moderate pH changes observed suggest that both fungal strains adapt their metabolism to maintain an environment conducive to enzymatic function. Thus, pH serves both as a regulator and an indicator of degradation efficiency in fungal-mediated polyethylene biodegradation.

### 3.2. Fourier Transform Infrared Spectroscopy

**Figure 4:**
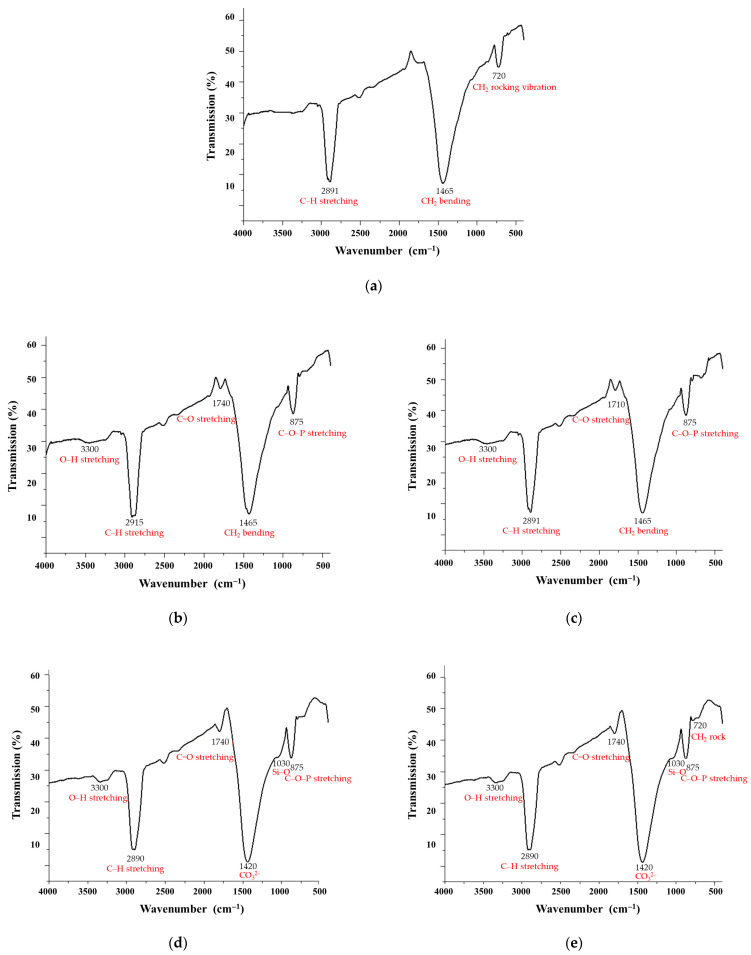
FTIR spectra of the polyethylene samples studied. Control polyethylene sample (**a**); polyethylene treated with *Aspergillus niger* in liquid medium (**b**); polyethylene sample treated with *Trichoderma harzianum* in liquid medium (**c**); polyethylene sample treated with *Aspergillus niger* in soil (**d**); and polyethylene sample treated with *Trichoderma harzianum* in soil (**e**).

The FTIR spectra of polyethylene samples subjected to fungal degradation by *A. niger* and *T. harzianum* in liquid and soil media revealed significant structural alterations, confirming microbial-induced polymer breakdown (Figure 4). The control sample exhibited the characteristic FTIR spectrum of pure polyethylene, with no significant oxidative or microbial degradation features. The control spectrum revealed high-intensity C–H stretching peaks in the 2800–3000 cm^−1^ region, indicative of aliphatic hydrocarbons in the complex molecular structure of polyethylene. Additionally, peaks occurred at 1465 cm⁻^1^ corresponding to CH_2_ bending (scissoring) vibration, indicative of the polymer’s methylene backbone and at 720 cm⁻^1^ indicating CH_2_ rocking vibration, a marker of crystalline regions in LDPE. The observed stability of the control sample implied that all observed changes in the treated samples were due to fungal activity, not abiotic factors.

Polyethylene samples treated with fungal cultures exhibited significant spectral alterations, confirming microbial-induced polymer breakdown. Notably, new absorption bands appeared in the regions of 1710–1740 cm⁻^1^ and 3300–3500 cm⁻^1^, corresponding to carbonyl (C=O) and hydroxyl (O–H) groups, respectively, indicative of oxidative degradation processes. Samples incubated in liquid media showed more pronounced oxidative features, with stronger C=O peaks and greater reductions in aliphatic C-H stretching intensities (2915–2848 cm⁻^1^), suggesting enhanced chain scission due to homogeneous fungal exposure and higher enzymatic activity. Conversely, soil-mediated degradation proceeded more slowly, exhibiting weaker oxidative peaks, likely due to restricted oxygen diffusion and interactions with soil minerals. However, unique spectral features such as a phosphoester band around 875 cm⁻^1^ were observed in *T. harzianum*-treated samples, indicating possible phosphate metabolism involvement. Both fungal strains preferentially targeted the amorphous regions of LDPE, leading to a reduction in the 720 cm⁻^1^ peak, signifying a loss of crystallinity. *A. niger* demonstrated a more aggressive oxidative degradation pattern, while *T. harzianum* exhibited stronger interactions with soil components, potentially through phosphate-related pathways. These results highlight how degradation efficiency and pathways depend on both fungal species and environmental conditions, with liquid systems favoring rapid, uniform breakdown, and soil environments promoting complex mineral–microbe–polymer interactions that may enhance long-term bioremediation potential.

### 3.3. Thermogravimetric Analysis

**Figure 5:**
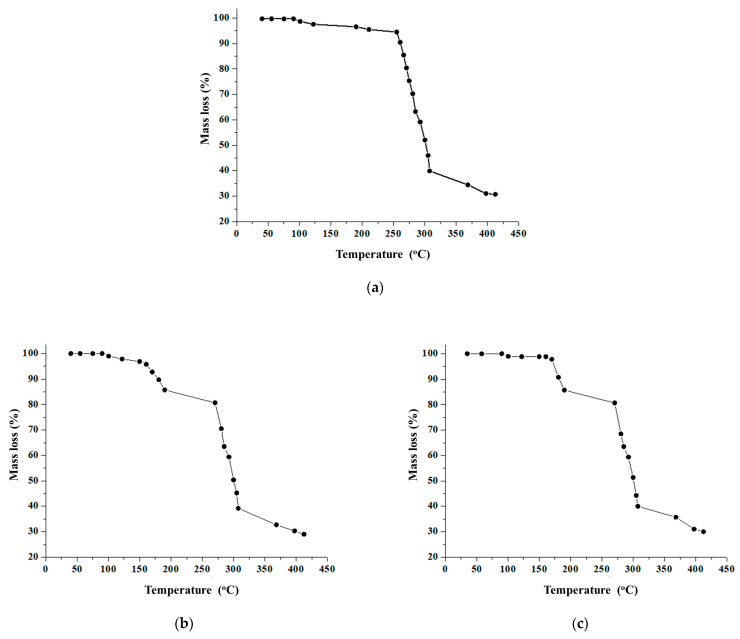

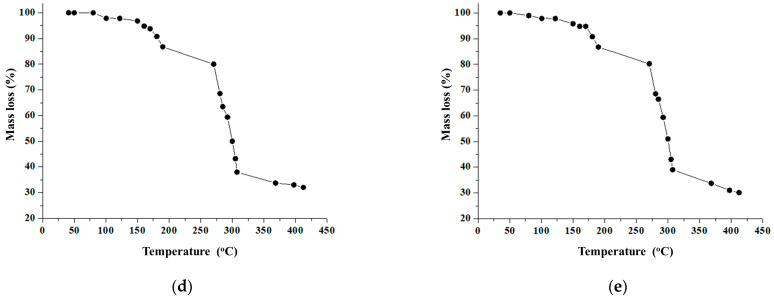
TGA curves of the polyethylene samples studied. Control polyethylene sample (**a**), polyethylene treated with *Aspergillus niger* in liquid medium (**b**), polyethylene sample treated with *Trichoderma harzianum* in liquid medium (**c**), polyethylene sample treated with *Aspergillus niger* in soil (**d**), polyethylene sample treated with *Trichoderma harzianum* in soil (**e**).

TGA was conducted to evaluate the thermal behavior of polyethylene before and after incubation with the fungal cultures (Figure 5). The control sample exhibited higher thermal stability compared to the fungal-treated samples. Specifically, the mass of the control sample remained stable up to approximately 200 °C, with significant mass loss commencing above 250 °C, consistent with the typical degradation onset of polyethylene in inert atmospheres, and decreased with increasing temperature. In contrast, initial degradation peaks have been observed in the biodegraded samples in the temperature range of 150 °C to 200 °C. This earlier onset of thermal degradation is attributed to the fungal cleavage of polymer chains in the degradation assembly, thus making the polyethylene sample less thermally stable than the control sample. Therefore, the decreased thermal stability also indicated that the fungal strains were metabolically active [70].

### 3.4. Scanning Electron Microscopy

**Figure 6:**
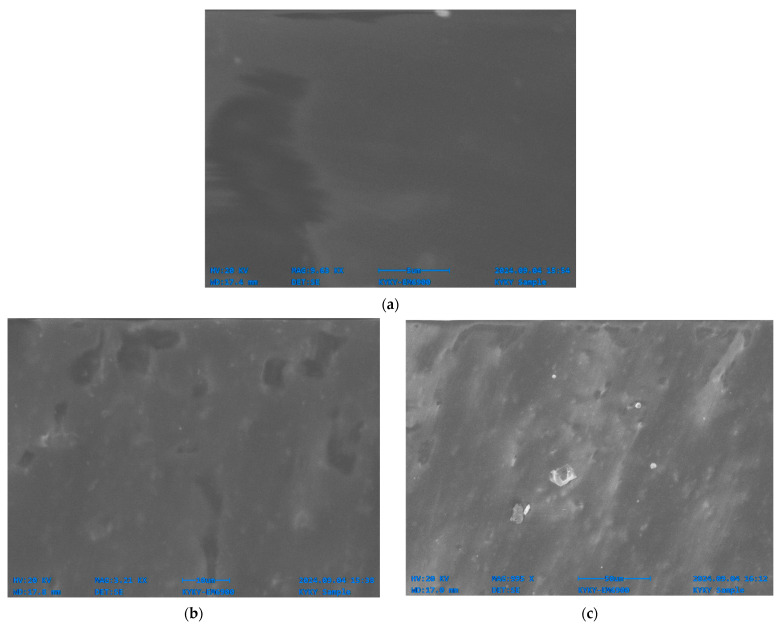

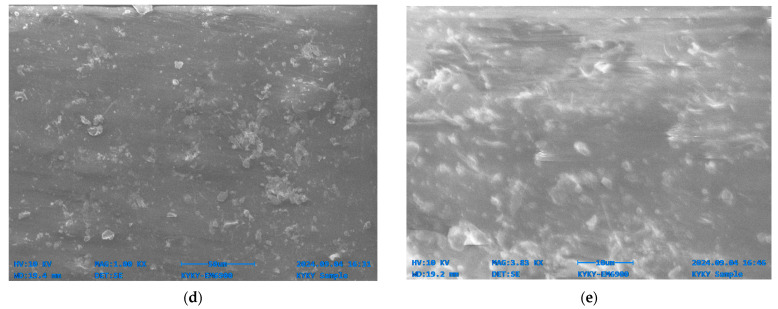
Scanning electron micrographs of polyethylene (PE) films (1000×) control sample (**a**); cultured with *Aspergillus niger* in liquid medium (**b**); *Trichoderma harzianum* in liquid medium (**c**); *Aspergillus niger* in soil (**d**); and *Trichoderma harzianum* in soil (**e**).

The surface morphology of polyethylene samples was examined using SEM, providing insights on the microscopic breakdown of polyethylene by fungi and helping visualize structural changes like pitting, roughening, and microbial colonization (Figure 6). The surface of polyethylene plastics is uniformly smooth and free of apparent damages or microbial activity prior to fungal exposure (Figure 6a). The stability of control samples throughout the experimental period confirmed that all observed physicochemical changes were exclusively attributable to fungal activity, ruling out any significant contribution from abiotic factors such as oxidation or environmental weathering. However, after exposure to fungal cultures, SEM images show notable changes: that plastic is colonized by fungal hyphae and mycelia, the surface becomes roughened with pits and grooves, and parts of the material degrade via enzymatic cleavage. The polymer chains are broken down by these fungal enzymes, reducing the molecular weight and creating porous, cracked regions. The general surface structure deteriorates, indicating the microbial breakdown of polyethylene and demonstrating the environmental potential of fungi to decompose synthetic polymers.

## 4. Discussion

The weight of the polyethylene film was measured before and after incubation with fungal cultures to assess the biodegradation of LDPE. It is hypothesized that the fungi altered the films by secreting enzymes that break down the material, utilizing polyethylene as a carbon source for their growth. This enzymatic activity resulted in a significant reduction in the weight of the films, consistent with findings in the scientific literature [26]. As shown in Table 1, the weight loss reduction of LDPE films in this study was 45.2 ± 0.21% for *A. niger* in liquid medium and 9.09 ± 0.08% in soil, while *T. harzianum* exhibited a 36.0 ± 0.21% weight loss in liquid medium and 10.00 ± 0.18% in soil. These results indicate that both fungal strains effectively degraded the polyethylene films by utilizing nitrogen and carbon for their growth. However, as reported in the scientific literature [71], the decomposition rates were still far from complete biodegradation. The lower efficiency observed in soil is likely due to the superior growth conditions provided in the liquid medium, highlighting the importance of environmental factors in the biodegradation of polyethylene films. A consistent decrease in the pH of the medium was also observed during the biodegradation process, as also reported by Das et al. [19] during the biodegradation of polyethylene by the *Aspergilli* (*A. niger*, *A. ornatus*, *A. nidulans*, *A. cremeus*, *A. flavus*, *A. candidus*, and *A. glaucus*) and *Fusarium* species. Those authors attributed this acidification to the presence of distinct monomer products during polymer degradation. Furthermore, fungi possess complex metabolic processes closely associated with the biodegradation of LDPE. These metabolic processes, which alter the pH of the culture media, are primarily responsible for the biodegradation of LDPE [42]. The culture media may become slightly acidic due to the strains’ potential to produce trace amounts of organic acids during the breakdown of LDPE. Numerous studies have demonstrated that fungi are more effective at degrading polymers in acidic environments [43,47,72]. Other investigations related to the fungal breakdown of polyethylene have reported that pH can drop to more acidic levels, as low as 4 [45]. Organic acids, such as oxalic acid, produced by *A. niger* acidify the culture medium or other environments [73] and promote the oxidation and enzymatic (lipase) cleavage of polyethylene [74,75], whereas *T. harzianum* favors neutral and less shift in pH due to biofilm-based breakdown [36,76]. Thus, changes in pH play a critical role in the degradation of LDPE by fungal strains.

The results of FTIR spectroscopy indicate distinct peaks in the treated samples compared to the control sample. Specifically, the peaks in the region of 2800–3000 cm⁻^1^ showed decreased intensity in all treated samples, which corresponds to the breakdown of aliphatic –CH_2_ bonds [77]. Additionally, new peaks formed in the region around 1700 cm⁻^1^, which were absent in the control sample. These peaks are indicative of aldehyde and ketone formation, which are intermediates produced during polyethylene biodegradation. New bands were also observed around the fingerprint region and between 875 cm⁻^1^, reflecting alterations in the molecular structure of polyethylene and indicating –CH bond deformation in the treated samples [28]. All spectral analyses show variations in peak intensities and the emergence of new peaks compared to the control sample, confirming that biodegradation has occurred in the fungal-treated samples. Notably, the *A. niger* treated samples in liquid medium exhibited greater peak variations than those treated with *T. harzianum.* Conversely, samples treated with *T. harzianum* in the soil medium showed more pronounced peak variations compared to those treated with *A. niger.*

To assess the thermal stability of polyethylene samples, TGA analysis was conducted. This analysis was essential to understand how fungal cultures degraded polyethylene samples relative to control samples by examining differences in their molecular weight [78]. Experimental conditions (sample size, nitrogen atmosphere, temperature range) were chosen in accordance with the scientific literature [68,69]. The mass loss measured by TGA at 412 °C was 29.01% for the sample treated with *A. niger* and 30.01% for that treated with *T. harzianum* in liquid medium. In the soil assays, the treated polyetylene samples from *A. niger* and *T. harzianum* exhibited mass losses of 32.00% and 30.01%, respectively, at the same temperature. The greater mass loss observed by TGA after incubation with *A. niger* in liquid medium compared to *T. harzianum* suggests that *A. niger* is more effective at degrading LDPE in this environment. In contrast, the soil assembly showed a higher TGA mass loss for *T. harzianum.* Thus, the degradation of the polymer is evident from the shifts in weight loss observed in TGA [79].

SEM analysis was used to further investigate the deterioration of LDPE films. Cracks, scions, and holes on the LDPE films’ surface indicated the degree of degradation. Similarly to cases reported in the scientific literature [31], the selected fungal strains degraded the intricate structure of LDPE into their monomeric forms as validated by the SEM images. By using the films as the only carbon source, the fungal strains were able to colonize the LDPE film. The fungal hyphae were able to successfully merge with the LDPE film by spreading out throughout its surface. Similar SEM analyses showed structural alterations and erosions on the surface of LDPE films, including porosity, cavities, holes, scions, and cracks caused by fungal consortia such as *Aspergillus* and other fungus species [80]. In our hands, when LDPE films were cultured with the selected fungal cultures, their smooth surfaces turned into holes and fissures. The fungi also produced a decrease in molecular weight and an increase in carbonyl double bond groups.

Although the study was unavoidably somewhat hindered by its inherent limitations posed by the experimental design, e.g., relatively short incubation periods, the potential influence of LDPE pretreatment, or the fact that simulated conditions may not fully represent natural environments, the results revealed the characteristic features of the degradative potential of the fungal strains studied. Because *Trichoderma harzianum* and *Aspergillus niger* have different ecological adaptations and enzymatic methods, they degraded polyethylene differently. The superior LDPE degradation by *A. niger* in liquid media likely stems from its prolific secretion of oxidative enzymes (e.g., lipases and esterases) under controlled conditions, while *T. harzianum’s* adaptability to soil driven by hyphal colonization traits and the siderophore-mediated nutrient acquisition explains its marginally higher efficacy in this environment. The observed differences underscore the importance of tailoring fungal-based remediation strategies to habitat-specific conditions, such as optimizing *A. niger* for wastewater treatment or leveraging *T. harzianum* for soil applications.

## 5. Conclusions

The findings of this study demonstrate the potential of the selected fungal strains to degrade polyethylene under the conditions that closely mimic real-world environments. By simulating soil and aquatic systems, the present study demonstrated the practical applicability of fungal-based biodegradation strategies, offering a sustainable and eco-friendly solution to the global challenge of plastic pollution. The use of analytical methods provided crucial insights into the degradation process, revealing that biodegradation, though gradual, was more efficient in salt medium compared to soil. Notably, *A. niger* exhibited greater degradation in liquid medium, while *T. harzianum* was more effective in soil which lays the foundation for customized fungal-based bioremediation strategies that use *Trichoderma harzianum* for soil contamination and *Aspergillus niger* for aquatic systems. Future work should explore synergistic fungal consortia to combine *A. niger’s* enzymatic efficiency with *T. harzianum’s* soil adaptability, optimize environmental parameters (pH, nutrients), and characterize the specific enzymes involved and explore genetic engineering to enhance degradation rates, and validate these processes at the pilot scale to bridge the gap between laboratory findings and real-world waste management applications. Such advancements could transform this approach into a viable, eco-friendly solution for mitigating persistent plastic pollution across diverse ecosystems.

## Figures and Tables

**Table 1:** Weight loss measurement of samples (n = 3, mean ± SD).

Medium	Fungal Strain $Weight\;loss\%=\frac{M_{0}-M_{f}}{M_{0}}\times 100$		*p*-Value	Significance
	*Aspergillus niger*	*Trichoderma harzianum*		
Liquid medium	45.62 ± 0.21	36.01 ± 0.21	*p* < 0.0001	****
Soil	9.09 ± 0.08	10.00 ± 0.18	*p* < 0.01	**

**** *p* < 0.0001, ** *p* < 0.01 (two-tailed *t*-test vs. untreated control).

## Data Availability

The data presented in this study are contained within the manuscript.
